# Obesity and periodontitis: a comprehensive review of their interconnected pathophysiology and clinical implications

**DOI:** 10.3389/fnut.2024.1440216

**Published:** 2024-08-07

**Authors:** Claudia Reytor-González, Juan Marcos Parise-Vasco, Natali González, Alison Simancas-Racines, Raynier Zambrano-Villacres, Ana Karina Zambrano, Daniel Simancas-Racines

**Affiliations:** ^1^Facultad de Ciencias de la Salud Eugenio Espejo, Centro de Investigación en Salud Pública y Epidemiología Clínica (CISPEC), Universidad UTE, Quito, Ecuador; ^2^Facultad de Odontología, Universidad UTE, Santo Domingo, Ecuador; ^3^Carrera de Medicina Veterinaria, Facultad de Ciencias Agropecuarias y Recursos Naturales, Universidad Técnica de Cotopaxi, Latacunga, Ecuador; ^4^Universidad Espíritu Santo, Samborondón, Ecuador; ^5^Facultad de Ciencias de la Salud Eugenio Espejo, Centro de Investigación Genética y Genómica, Universidad UTE, Quito, Ecuador

**Keywords:** obesity, periodontitis, oxidative stress, inflammatory response, periodontal treatment

## Abstract

Obesity and periodontitis are significant health problems with a complex bidirectional relationship. Excess body fat is linked to systemic diseases and can lead to persistent inflammation, potentially harming periodontal health. Periodontitis, a chronic inflammatory condition affecting the supporting structures of teeth, poses substantial health risks. Both conditions share pathological processes such as inflammation and oxidative stress, which aggravate health status and make treatment more challenging. Understanding this interaction is crucial for developing effective management strategies for both diseases. This study explores the multifaceted aspects of obesity and periodontitis and their reciprocal relationship.

## Introduction

Obesity and periodontitis are serious public health issues that increase the burden of general health and chronic illnesses (1–3). Obesity, characterized by the abnormal accumulation of body fat, is linked to comorbidities such as insulin resistance, cardiovascular diseases, and certain cancers (1, 4). It induces a low-grade chronic inflammatory state, releasing proinflammatory mediators that may link it to periodontitis (5, 6).

Periodontitis is a chronic inflammatory disease caused by microbial-host interactions. It destroys tissue by affecting the supporting structures of teeth (7, 8) and impacts overall wellbeing (9).

The bidirectional relationship between obesity and periodontitis is complex and multifaceted. Adipose tissue functions as an endocrine organ, releasing cytokines, and proinflammatory hormones that contribute to systemic inflammation and oxidative stress—common pathophysiological mechanisms shared by both conditions (7). Epidemiological studies support the notion that obesity is a significant risk factor for the development and exacerbation of periodontitis (10–12). Likewise, several studies suggest that periodontitis may increase obesity-related disorders such as intestinal dysbiosis (13) and insulin resistance (14, 15).

Understanding the connection between obesity and periodontitis is crucial, as both conditions are highly prevalent worldwide. Examining their relationship not only has implications for oral health but may also reveal the mechanisms underlying a variety of systemic diseases, providing opportunities for preventive, and therapeutic interventions that could significantly improve the population’s overall health.

This narrative review explores the multifactorial aspects of obesity and periodontitis and their bidirectional relationship. It examines the interplay between these conditions, from inflammatory responses and oxidative stress to changes in periodontal microbiota and their impact during pregnancy or after bariatric surgery. Furthermore, the article delves into the implications of both non-surgical and surgical periodontal therapies in patients with obesity, emphasizing the need for comprehensive approaches to prevention and treatment.

Understanding the intricate connections between obesity and periodontitis is crucial for developing effective strategies to manage these interrelated conditions. As research continues to uncover the complexities of this relationship, healthcare practitioners can enhance their knowledge to provide more targeted interventions, ultimately improving the overall health outcomes of individuals affected by obesity and periodontitis.

## Methods

For this narrative review, we considered publications from 1977 to 2023. The search was conducted through PubMed and Cochrane Library, using a combination of related search terms, including “periodontitis,” “obesity,” “oxidative stress,” “inflammatory response,” and “periodontal treatment.” Three research team members (CR-G, JMP-V, and DS-R) reviewed the articles by titles and abstracts, selecting them for full review only if all authors agreed on their relevance. Additionally, the research team examined the references from the identified articles to incorporate additional relevant publications. Ultimately, we reviewed 33 observational studies, seven cohort studies, three case–control studies, 20 systematic reviews, eight clinical trials, 63 reviews, and 13 studies with other designs, such as animal studies or conference reports. The chosen articles underwent a comprehensive content analysis to determine evidence of the relationship between periodontitis and obesity.

### Obesity

Obesity is a severe medical condition worldwide (1) characterized by excessive or abnormal accumulation of body fat, which increases the risk of several chronic diseases (3). It is primarily classified by body mass index (BMI), calculated as weight in kilograms divided by the square of height in meters (kg/m^2^), with obesity defined as a BMI of 30 or higher (16).

In the past three decades, the prevalence of obesity has increased at an alarming rate, with a 27.5% increase in adults and a 47.1% increase in children (4). The exact cause of obesity remains elusive; however, it appears to involve a complex interaction of biological, psychosocial, and behavioral factors, including genetic composition, metabolic disorders, physical inactivity, socioeconomic status, a high-calorie diet, and cultural influences (4, 17).

Obesity is associated with numerous comorbidities affecting almost all body systems, such as insulin resistance, type 2 diabetes mellitus, hepatic steatosis, cardiovascular disease, hypertension, cerebrovascular accidents, lipid metabolism disorders, gallbladder problems, osteoarthritis, sleep apnea, and other respiratory problems (1, 4, 18, 19). It is also linked to certain types of cancer, including breast, ovarian, endometrial, prostate, liver, gallbladder, kidney, colon, and thyroid cancers (1, 4, 20–23).

A key aspect of obesity is its role in inducing a state of low-grade chronic inflammation (24) and its association with inflammatory markers related to systemic disease (5, 6). In addition to storing energy, adipose tissue functions as an active endocrine organ, secreting various chemical mediators (25). These factors include leptin, cytokines such as tumor necrosis factor-alpha (TNF-α) and interleukins, adiponectin, complement components, plasminogen activator inhibitor-1, proteins of the renin-angiotensin system, and resistin (25–27). Some of these substances, like cytokines, play a critical role in systemic inflammation (5) and may serve as a link between obesity and other inflammatory conditions such as periodontitis (28).

### Periodontitis

Periodontitis is a chronic, non-communicable inflammatory disease that results from the interaction between pathogenic microorganisms and the host’s immune system (7). This condition destroys the tissues surrounding and supporting the tooth, including the gums, alveolar bone, and periodontal ligament (8), as a consequence of the release of proinflammatory mediators (29). The most common signs of this disease include gingival inflammation, loss of alveolar bone, dental mobility, increased probing depth, and gingival bleeding (2, 30).

The global oral health status report estimated that severe periodontal diseases affect approximately 19% of the global adult population, accounting for over 1 billion cases worldwide (9). This has made the disease a significant public health issue that causes disability, negatively impacts chewing and aesthetics, and reduces quality of life (2, 31).

According to the National Health and Nutrition Examination Survey of the United States, 42% of adults had periodontitis by 2014 (32, 33), indicating that although the disease can appear from the age of 15, its prevalence increases with age, with older adults being the most vulnerable group where more aggressive forms are presented (9, 30).

Various factors can disturb the natural balance in the mouth, leading to a shift in the biofilm beneath the gums towards proinflammatory dysbiosis. This imbalance involves excessive growth of microorganisms such as *Porphyromonas gingivalis*, *Tannerella forsythia*, and *Treponema denticola*, triggering chronic inflammation (34–36).

These bacteria colonize host tissues and evade defense mechanisms. *Porphyromonas gingivalis* fimbriae binds to other bacteria, such as *Treponema denticola*, and human proteins, such as glyceraldehyde-3-phosphate dehydrogenase, to facilitate adherence and invasion of host cells (37). The macromolecules that comprise the biofilms produced by these bacteria maintain proximity between bacterial and host cells, promoting health and disease (38).

They have also created several ways to obtain iron from the host environment, which is essential for their growth and contributes to biofilm dysbiosis (39). In addition, flagella-assisted motility allows these pathogens to seek nutrients and colonize favorable niches. At the same time, their metabolic activity and rapid growth enhance their ability to resist natural removal and mechanical debridement (38).

Another protective mechanism of these microorganisms is the production of capsules that prevent phagocytosis and release proteases that affect chemotaxis and neutrophil activation to evade host defense mechanisms. *Porphyromonas gingivalis* can also release outer membrane vesicles that scavenge interleukin-8 (IL-8), thereby protecting itself from host defense systems (40). In addition, bacteria such as *Porphyromonas gingivalis*, *Tannerella forsythia*, *Aggregatibacter actinomycetemcomitans*, and *Fusobacterium nucleatum* can invade host cells and escape the immune system (38).

Finally, bacterial exotoxins and endotoxins contribute to the virulence of these pathogenic species by damaging host cells and promoting the release of inflammatory cytokines. Enzymes, such as collagenases and gingipains from *Porphyromonas gingivalis*, destroy tissue components and host defense molecules (41).

This change in the microbiome can trigger periodontitis in susceptible individuals, characterized by an inadequate inflammatory response and the consequent destruction of connective tissue and alveolar bone (42, 43).

Periodontitis is a multifactorial disease. Various risk factors are associated with the onset of periodontitis that can affect the relationship between the host and microorganisms. Smoking is the most significant risk factor (44–47), along with metabolic diseases like diabetes mellitus (48–51), obesity (7, 10, 52), stress (53, 54), genetic factors (55), and oral hygiene habits (56).

### Inflammatory response

Inflammation is the immune system’s biological response to organic, chemical, or physical stimuli to protect living organisms from harmful factors, including fungi, viruses, and bacteria (57). In its controlled form, as in acute inflammation, this process is crucial in eliminating pathogens, cellular debris, and inflammatory mediators while stimulating tissue repair. This leads to the resolution of inflammation and the restoration of tissue homeostasis (57, 58).

In the acute phase of the inflammatory response, immune system cells, including platelets and granulocytic cells such as basophils, mast cells, neutrophils, and eosinophils, become activated and subsequently produce and release a variety of chemical mediators, including cytokines, chemokines, and acute-phase proteins (59). These substances promote vasodilation and increase vascular permeability, facilitate the migration of immune cells to the site of inflammation, and stimulate and regulate the inflammatory response (59, 60). Depending on the extent of the injury, this acute phase may be sufficient to resolve the damage (61).

Conversely, failure to resolve inflammation and persistent inflammation, either as a result of prolonged exposure to a stimulus or a persistent pathogen, non-degradable foreign bodies, or an inappropriate autoimmune response against self-cells, can lead to the chronic phase of inflammation in which tissue damage (60, 61), fibrosis and granuloma formation can occur (60). The mechanisms involved in chronic inflammation contribute to the development of many diseases, including arthritis, asthma, atherosclerosis, autoimmune diseases, type 2 diabetes mellitus, cystic fibrosis, inflammatory bowel disease, Parkinson’s disease, Alzheimer’s disease, cardiovascular diseases, cancer, and conditions associated with aging (57, 61, 62).

In obesity, chronic inflammation is marked by elevated levels of pro-inflammatory cytokines such as TNF-α, interleukin-1 beta (IL-1β), and interleukin-6 (IL-6), primarily produced by adipose tissue-derived macrophages (63), and by the adipose tissue itself, as previously mentioned (25). Furthermore, various factors currently under investigation can exacerbate the inflammatory process. Among these, non-esterified fatty acids may induce inflammation through mechanisms such as modulation of adipokine production or activation of Toll-like receptors; excess nutrients and adipocyte expansion can cause endoplasmic reticulum stress; and hypoxia in hypertrophied adipose tissue could stimulate the expression of inflammatory genes and activate immune cells (64). In contrast, in periodontitis, chronic inflammation originates from a complex immune response triggered by persistent microbial elements in the oral cavity, causing local damage and systemic effects (65), suggesting a potential interaction with the systemic inflammation observed in obesity (28).

### The bidirectional relationship between obesity and periodontitis

The intricate connection between obesity and periodontitis has emerged as a crucial research area in periodontal medicine. Adipose tissue, acting as an endocrine organ, releases cytokines and proinflammatory hormones, known as adipocytokines, triggering inflammatory processes and oxidative stress disorders (7, 29, 66). This generates a shared pathophysiology between both diseases. Explored through epidemiological studies and clinical trials, this link reveals a bidirectional relationship between obesity and periodontitis (10–12), where exacerbated proinflammatory factors worsen the severity of both conditions.

Since the early reports of the relationship between obesity and periodontitis in animals in 1977 (67) and in humans in 1998 (66), numerous studies have supported the hypothesis that obesity constitutes a risk factor for the development and worsening of periodontitis. Epidemiological research results indicate that individuals with obesity show a higher prevalence of periodontal disease compared to the normal-weight population (11). Furthermore, the strength of this correlation seems to intensify with an increase in obesity (11, 12).

During obesity, adipose tissue increases, and adipocytes secrete fewer anti-inflammatory substances, such as adiponectin, while increasing the secretion of proinflammatory substances, such as leptin and chemokines (68). This leads to an infiltration of immune cells, likely early arrivals being B and T cells, influencing the secretion of proinflammatory cytokines and Interferon gamma (IFN-γ), essential for activating macrophages and inflammation. Inflammation in obesity is characterized by the abnormal presence of these cytokines, which may hinder the elimination of pathogenic microorganisms in the oral cavity (69). And induce the destruction of characteristic periodontal connective tissue and bone (70).

Inflammatory biomarkers such as IL-1, IL-6, TNF-α, and matrix metalloproteinases (MMP) (63) play a crucial role in the relationship between obesity and periodontitis (71). Elevated levels of these biomarkers, commonly associated with obesity, correlate with losing the extracellular matrix, inhibiting osteoblastogenesis, and activating osteoclasts, leading to collagen and bone destruction (8, 72).

**Figure 1:**
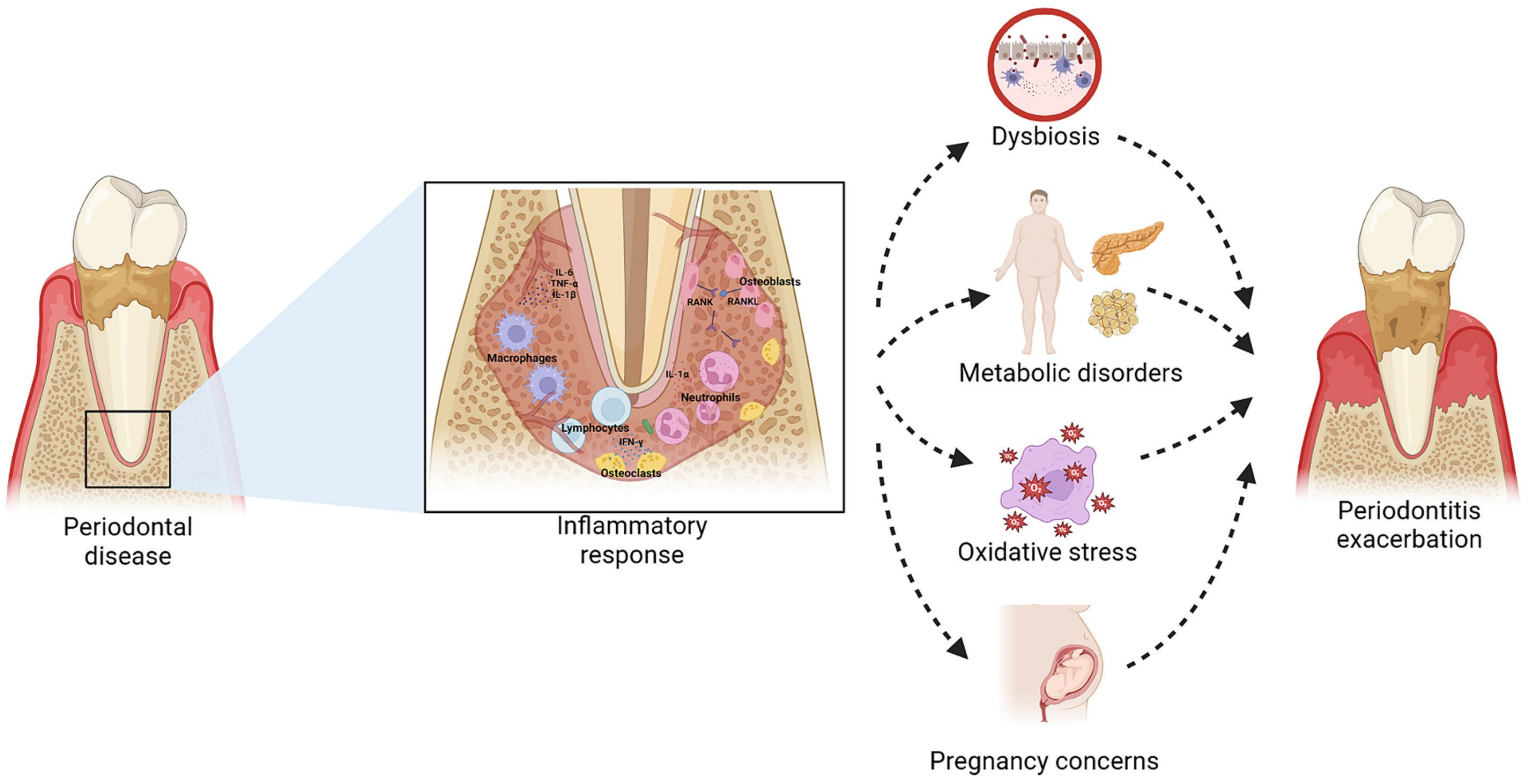
Relationship between obesity and periodontitis. Inflammation in periodontal disease, mediated by the release of cytokines such as IL-6, TNF-α, and IL-1β, can be exacerbated in individuals with obesity due to a systemic proinflammatory state. This inflammatory condition contributes to dysbiosis and oxidative stress, worsening periodontitis. Additionally, chronic periodontal inflammation can negatively influence metabolic disorders and increase the risk of pregnancy complications, perpetuating a negative feedback cycle that impacts both oral and systemic health. Created with BioRender.com.

Several studies have analyzed the cytokine profile in the crevicular fluid of patients with and without obesity and chronic periodontitis. Some have reported significantly higher levels of these proinflammatory substances in patients with obesity (73–76). Others show no differences between these two groups (77–79), highlighting the need for further analysis of the effects of obesity control on the cytokine profile in crevicular fluid and other fluids of patients with obesity and periodontal disease (71) (Figure 1).

Our understanding of these findings enables us to deduce that obesity and periodontitis are related. That being said, more research is necessary to ascertain whether these two disorders are causally related.

### Obesity and bone loss

Initially, it was believed that obesity stimulated bone formation (80), but now the available evidence supports that obesity induces changes in bone density and affects periodontal health (81).

The increase of fatty tissue in the bone marrow acts as an endocrine organ that secretes various pro-inflammatory adipokines such as leptin and resistin while decreasing the secretion of anti-inflammatory substances such as adiponectin (82). These pro-inflammatory adipokines induce a chronic low-grade inflammatory state characterized by the elevation of inflammatory biomarkers such as TNF-α and IL-6, which increase osteoclastic function and reduce osteoblast formation—leading to increased bone resorption and decreased bone mineral density (83–85).

Similarly, obesity can trigger changes in the intestinal microbiota, affecting bones, including the jaw, through pathobionts or circulating metabolites that stimulate bone resorption (86).

On the other hand, studies addressing the relationship between obesity and alveolar bone loss are scarcer but also present obesity as an established risk factor for periodontitis (10). Several animal studies have reported that obesity and dyslipidemia (87), as well as a diet high in carbohydrates and palmitic acid (88, 89), contribute to increased bone loss in *Porphyromonas gingivalis*-induced periodontitis (89). This includes deterioration of trabecular bone architecture, decreased cortical bone density in the alveolar bone area, and increased serum leptin levels (90).

Another significant finding is that individuals with obesity are more susceptible to alveolar bone loss, clinical attachment loss, and, consequently, edentulism (12) compared to those without obesity (91). Obesity-induced systemic inflammation may interfere with eliminating pathogenic microorganisms in the oral cavity, promoting the destruction of periodontal connective tissue and alveolar bone. The release of proinflammatory cytokines and oxidative stress contribute to the progression of periodontitis in individuals with obesity, exacerbating the destruction of periodontal tissue (82). In addition, factors such as subgingival calculus, probing depth greater than 4 mm, and bleeding on probing are more frequent in patients with obesity (92), suggesting that obesity could be a significant risk factor, even in patients with clinically healthy periodontium (93).

These mechanisms underscore the need for a comprehensive approach to address obesity, bone density, and periodontal health.

### Oxidative stress

Oxidative stress is an imbalance between reactive oxygen species (ROS) and the body’s antioxidant systems, causing damage to proteins, lipids, and DNA (94). This condition can act as a defense mechanism of the immune system against the presence of bacteria, such as those causing periodontitis (95). After periodontal pathogenic bacteria trigger host defense responses in the biofilm, neutrophils become the most common inflammatory cells in the periodontal tissue and gingival crevice. Neutrophils are believed to be the primary sources of ROS in periodontitis (96).

The interplay between periodontitis, obesity, and oxidative stress is a significant area of study that highlights the complex interactions contributing to chronic inflammatory conditions. Oxidative stress exacerbates both conditions, leading to cellular and tissue damage (97).

Recent studies have shown that oxidative stress plays a crucial role in the pathogenesis of both periodontitis and obesity (98). Excessive adipose tissue in individuals with obesity increases ROS production, which induces oxidative damage in gingival tissues, contributing to periodontal destruction and alveolar bone loss. This oxidative damage is more pronounced in patients with obesity compared to those of average weight, indicating a strong link between obesity and periodontal oxidative stress (97, 99).

Another study highlighted higher oxidative stress markers, such as myeloperoxidase and nitric oxide, in the gingival crevicular fluid of individuals with obesity and periodontitis. These markers are associated with increased inflammation and tissue destruction in periodontal disease (97). Additionally, the study found that non-surgical periodontal therapy significantly reduced these oxidative stress markers, suggesting that periodontal treatment can mitigate oxidative damage and improve periodontal health in patients with obesity (97, 100).

**Figure 2:**
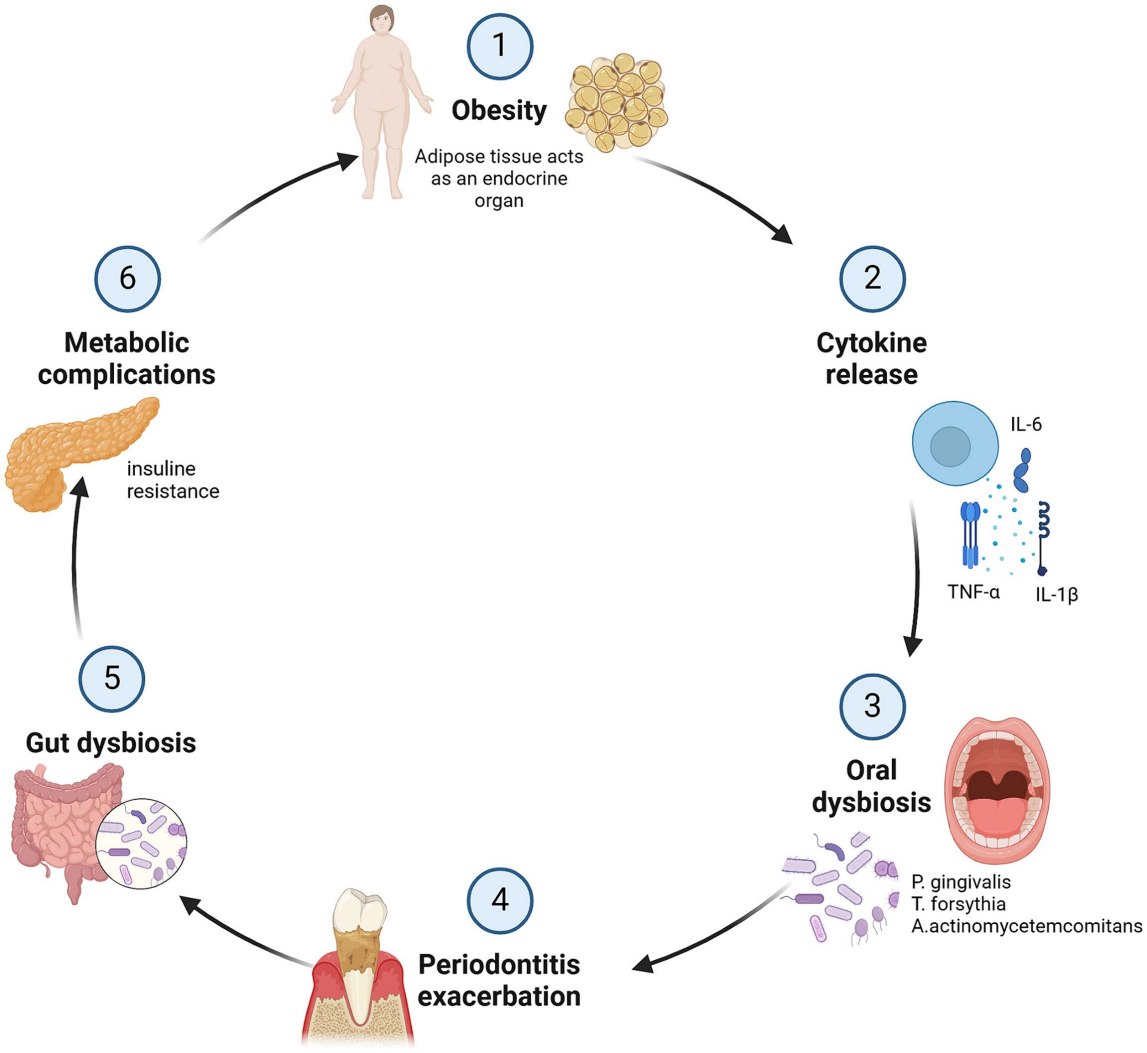
Impact of obesity on periodontal inflammation: a bidirectional cycle of damage. In obesity, adipose tissue acts as an endocrine organ releasing inflammatory substances such as TNF-α, IL-1β, and IL-6, leading a dysbiosis that contributes to periodontal inflammation and exacerbation of periodontitis, resulting in the destruction of periodontal tissue and bone loss. Chronic inflammation is also associated with metabolic complications like insulin resistance, creating a bidirectional cycle of inflammation and damage between obesity and periodontitis. Created with BioRender.com.

Evidence also suggests that periodontitis can influence systemic oxidative stress, causing a sustained inflammatory response that may contribute to insulin resistance, a common phenomenon in obesity (99). This resistance can affect glucose metabolism and appetite regulation, contributing to weight gain (97) (Figure 2).

This interaction underscores the need for comprehensive therapeutic approaches addressing periodontal and systemic health. Periodontal therapy and lifestyle modifications can mitigate the adverse effects of these chronic conditions by reducing oxidative stress and managing inflammation.

### Periodontal microbiota

The periodontal microbiota and obesity are closely related through a process of dysbiosis, an alteration in the composition of the oral microbiome that can exacerbate periodontitis and be influenced by the individual’s obesity status.

Periodontitis is characterized by a dysbiotic oral microbiome characterized by an increase in periodontal pathogens such as *Porphyromonas gingivalis*, *Aggregatibacter actinomycetemcomitans*, and *Tannerella forsythia* (101). In patients with obesity, a higher prevalence and severity of periodontitis are observed, which is related to an altered microbial composition in the oral cavity (102, 103).

Obesity contributes to the dysbiosis of the subgingival microbiome due to several factors, including systemic inflammation and altered immune response. Excess fatty tissue in individuals with obesity produces inflammatory mediators and ROS, affecting systemic metabolism and periodontal health. Several studies have reported an increase in the proportion of *Tannerella forsythia* in subgingival plaque and *Porphyromonas gingivalis* in the saliva of patients with obesity compared to those without obesity (86), which exacerbates gingival inflammation and reduces the effectiveness of periodontal treatment in these patients (103, 104).

Conversely, periodontal inflammation can also contribute to systemic inflammation (104, 105), exacerbating obesity and its metabolic complications, such as insulin resistance and chronic inflammation, which are common in obesity (106). Periodontal inflammation can contribute to intestinal dysbiosis (107), creating a vicious cycle perpetuating poor oral and systemic health (108). This bidirectional link underscores the importance of addressing oral health and obesity in an integrated manner to improve clinical outcomes.

Interventions such as periodontal therapy and lifestyle modifications are crucial to breaking this cycle of dysbiosis and inflammation. Including dietary strategies, regular exercise, and reasonable oral hygiene control can help restore microbial balance and reduce the impact of obesity on periodontal health.

### Periodontitis in pregnant women with obesity

Obesity and periodontitis are both health concerns that interact in complex ways, particularly affecting pregnant women. During pregnancy, women undergo significant hormonal, immunological, and metabolic changes essential for proper fetal development and the provision of blood, nutrients, and oxygen (109). These changes and high hormone levels impair connective tissue regeneration in the periodontium, increasing the inflammatory response in these tissues. This phenomenon may increase the proliferation of aerobic and anaerobic bacteria, thereby raising the prevalence of pregnancy-related periodontal disease (109, 110).

Maternal obesity further complicates this scenario by inducing systemic immunological and inflammatory changes that may exacerbate pregnancy’s inherent inflammatory state (111). This altered immune response can increase susceptibility to infections and excessive immunological reactivity, influencing the severity of maternal periodontitis (112).

Several studies have shown a positive association between obesity and periodontal disease (109–111, 113, 114), suggesting that both conditions may synergistically increase the inflammatory and oxidative state in pregnant women. This is reflected in an increase in local and systemic biomarkers (111) and could lead to an increase in complications associated with maternal obesity, such as gestational diabetes mellitus, hypertension, placental abnormalities, pre-eclampsia, prematurity, fetal death, and spontaneous abortion (109, 111). These adverse outcomes are believed to be linked to direct and indirect mechanisms involving periodontal pathogens and systemic inflammation. Direct mechanisms involve the translocation of oral bacteria to the placenta, triggering inflammatory responses, while indirect mechanisms involve elevated systemic inflammatory cytokines that disrupt placental function (115, 116).

Although there is evidence of an association between obesity and periodontal disease during pregnancy, the certainty of the evidence for these associations and their implications is inconclusive. This is due to current studies’ methodological, clinical, and statistical heterogeneity, a potential risk of bias, and a lack of control for confounding factors. Therefore, new studies with research designs that use rigorous methods that minimize the risk of bias are needed to gain a better understanding and accuracy of these associations and their clinical implications.

### Periodontitis in bariatric surgery patients

There are multiple types of bariatric surgery, the most common being gastric bypass, sleeve gastrectomy, and adjustable gastric banding (117). Regardless of the type of surgery performed, these surgical procedures are superior to non-surgical interventions in terms of weight loss outcomes and improvement in obesity-related comorbidities (118).

Studies investigating the relationship between bariatric surgery and periodontitis yield mixed results. On the one hand, some studies suggest that surgery is associated with improvements in various metabolic and physiological aspects of the body, including improvements in periodontal health due to a reduction in the inflammatory state and adipose tissue burden (119–121), as well as improved control of dental biofilm (120, 121). One study found no apparent reduction in periodontitis after bariatric surgery but noted that malabsorption of critical nutrients could affect periodontal health (122). Meanwhile, two cohort studies (123, 124) and a systematic review suggest that periodontal status may worsen in the first 6 months after bariatric surgery (125). Therefore, it is recommended to conduct periodontal evaluations and appropriately manage oral health before undergoing surgical interventions to prevent further deterioration of periodontal health post-surgery (123–125).

### Non-surgical periodontal therapy in patients with obesity

The therapeutic approach to periodontitis encompasses various strategies, among which fundamental clinical interventions such as scaling and root planing stand out and are recognized as one of the pillars of non-surgical periodontal therapy. This treatment involves the meticulous removal of tartar and impurities from the root surfaces of teeth with a probing depth ≥5 mm (126).

Several studies have evaluated the effect of periodontal scaling and root planing on gingival bleeding, probing depth, and cytokine levels in patients with and without obesity and chronic periodontitis (127). While most research reports greater probing depth and higher levels of IL-1β, IL-6, TNF-α, IFN-γ, leptin, adiponectin, and CRP in patients with obesity compared to those with normal weight (127–130), the effects of periodontal therapy are inconclusive. Subgroup analysis in specific studies has provided a deeper insight into how obesity and periodontitis interact. In some instances, treatment decreases serum levels of proinflammatory substances in patients with obesity. Still, after 3 months of follow-up, high levels of IL-6 and tumor necrosis factor-α are observed in this patient group (131). Resistin, another proinflammatory mediator, exhibits higher levels in individuals with periodontitis than those without the disease. Despite efforts of periodontal treatment, resistin shows no significant changes in serum or gingival crevicular fluid levels in individuals with and without obesity over time, indicating that its proinflammatory expression persists (127, 129, 131).

In the pharmacological realm, various studies assert that controlled administration of antibiotics can play a significant role in managing the bacterial load associated with periodontitis (132–135) and leads to significant improvement in treatment by reducing probing depth and enhancing clinical attachment (136). Specific case considerations guide the choice of antimicrobial agents, which can be administered systemically or locally (137, 138).

Long-term maintenance is an essential treatment component, involving regular clinical follow-up, periodontal evaluations, and periodic professional cleanings. Patient education, focusing on effective oral hygiene practices and understanding risk factors, strengthens the preventive component and contributes to the sustainability of therapeutic outcomes (126).

### Surgical periodontal therapy in patients with obesity

Regarding surgical periodontal therapy, there are currently no studies directly comparing the outcomes of surgical periodontal therapy with non-surgical treatment in patients with obesity. However, there is evidence suggesting that patients with obesity may experience slower healing due to an exacerbated inflammatory response (63, 71), which could affect the results of surgical interventions (63), including surgical periodontal treatment.

In addition, it is common for patients with obesity to have coexisting comorbidities that may complicate surgical periodontal therapy (72, 74). This intersection of health conditions highlights the need for a comprehensive and personalized approach to the periodontal management of these patients. Based on the available evidence, non-surgical periodontal therapy may be preferable to minimize postoperative morbidity in this patient population (73, 75–77).

## Discussion

The results of this review indicate that obesity and periodontitis are interrelated through inflammatory and oxidative stress mechanisms, generating a cycle where each condition may aggravate and perpetuate the other. Adipose tissue, acting as an endocrine organ, triggers inflammatory responses that affect periodontal tissues, and the chronic inflammation associated with periodontitis may contribute to the metabolic imbalances seen in obesity. However, the causal relationship between these two pathologies is unclear.

Many studies suggest that obesity is a significant risk factor for periodontitis and that there could be a dose–response relationship associated with body mass index (10, 129, 139, 140). However, other studies that consider the type of obesity only associate altered periodontal parameters with abdominal obesity and discard the relationship between general obesity and gingival attachment loss and bleeding (128, 141).

Another factor analyzed in this study was the level of cytokines present in patients with and without obesity and periodontitis. While there are studies that reported considerably high levels of IL-8, IL-1 β, TNF- α, progranulin, monocyte chemoattractant protein-4 (MCP-4), lipocalin, and resistin (73–76, 142), other investigations report no difference in the levels of these biomarkers in both subgroups (77–79). This variability in the results may be because the studies that reported comparable levels of pro-inflammatory substances in patients with and without obesity and periodontitis did not consider other factors such as systemic diseases, smoking, or the depth of periodontal probing.

It is also essential to evaluate cytokine and adipocytokine levels in different biological fluids, such as saliva, gingival crevicular fluid, and serum. While saliva and gingival crevicular fluid are more specific indicators of local periodontal conditions, serum provides a more comprehensive view of the organism (71). The choice of biological fluid can influence the interpretation of results, highlighting the need for comprehensive approaches in periodontal and obesity research.

The results related to the impact of obesity on periodontal treatment are diverse. Some authors suggest that clinical attachment levels and probing depth are comparable in subjects with and without obesity after non-surgical periodontal treatment (131, 143). At the same time, other investigations reported that patients with obesity have a lower response to periodontal therapy compared to those with normal weight (143–145), highlighting the negative effects of chronic inflammation on the periodontium. This variability calls for studies with higher methodological quality to evaluate the clinical impact of periodontal therapy in patients with obesity in the long term. Conversely, some studies indicate that periodontal treatment can improve the lipid profile (146), positively impacting obesity control.

Obesity and periodontal disease during pregnancy may also be associated. Still, the evidence is not definitive because of methodological and statistical heterogeneity, potential biases, and the inability of current research to control for confounding factors. More rigorous research is needed to clarify these associations and their clinical implications.

Regarding bariatric surgery, it has been reported that patients who lost weight after this intervention significantly improved periodontal health compared to those who did not undergo surgery (147). These results indicate that individualizing nutritional counseling, physical exercise for weight reduction, and periodontal therapy in this group of patients is imperative to improving oral and general health (147).

It should also be noted that evidence on the results of surgical periodontal therapy in patients with obesity is limited. There are no studies that directly compare the clinical effects of surgical and non-surgical periodontal treatment in patients with obesity, but the possible exacerbated inflammatory response in patients with obesity could influence the speed of healing and the results of surgical interventions, suggesting that non-surgical therapy could be preferable in this group (63, 71).

This review had certain limitations that must be considered. Firstly, the heterogeneity of the included study designs generates variability in the results, making it difficult to generalize the conclusions. Differences in study populations, methodologies, and outcome measures contribute to this heterogeneity. Additionally, the potential for various biases exists, such as selection bias, reporting bias, and confounding factors that were not consistently controlled across studies. These biases can affect the validity and reliability of the findings. The lack of control for confounding variables in observational studies significantly limits the ability to establish a causal relationship between both pathologies. Many studies did not report controlling for confounding factors like systemic diseases, smoking, dietary habits, and physical activity, which could influence the observed relationships.

Secondly, the scarcity of longitudinal designs also represents a weakness since the temporal dynamics in the relationship between obesity and periodontitis cannot be assessed. Longitudinal studies are essential to determine the directionality and causality of the observed relationship over time.

It is important to proceed cautiously when extrapolating these results. Most evaluated investigations were carried out in particular populations, frequently in specific geographical areas or clinical situations. Diverse populations possess varying genetic, environmental, and lifestyle components, which may impact the generalizability of the findings in broader settings. For example, dietary habits, socioeconomic status, and healthcare access can all significantly impact periodontal health and obesity.

Future studies should strive to include varied populations from various socioeconomic backgrounds and geographic locations to improve the generalizability of the results. They should also look at how these correlations appear in particular subgroups, such as older people and other ethnic groups, to create tailored interventions that take into account their specific requirements.

Despite the limitations, this review presents several strengths. The breadth of the research, addressing aspects ranging from inflammatory mechanisms to outcomes in specific groups such as pregnant women and patients undergoing bariatric surgery, provides a comprehensive view of the relationship between obesity and periodontitis. Additionally, analyzing multiple factors, such as the potential causal relationship and responses to different available treatments, enriches the understanding of the interaction between periodontitis and obesity.

Several directions for future research are suggested to advance the understanding of this relationship. Prospective and longitudinal studies with long-term follow-ups are essential to establish causality and comprehend temporal dynamics. Focusing on specific populations, such as pregnant women, patients after bariatric surgery, and the younger population, will allow for more targeted therapeutic approaches. Exploration of modifying factors like genetics and the environment can provide valuable information for personalized therapeutic strategies.

In the realm of clinical practice, the analysis of the relationship between obesity and periodontitis has significant implications. A comprehensive patient assessment, considering obesity as a risk factor in periodontal evaluation, is recommended, especially in more susceptible populations such as pregnant women. A multidisciplinary approach involving healthcare professionals, including dentists, nutritionists, and surgeons, may be essential for effectively managing oral and general health in patients with obesity. Furthermore, patient education on the relationship between obesity and periodontitis and maintaining healthy habits can enhance awareness and promote prevention.

## Conclusion

In conclusion, the relationship between obesity and periodontitis is multifaceted and complex, involving inflammatory and oxidative stress mechanisms. The evidence suggests that obesity significantly increases the risk of developing and exacerbating periodontitis, with elevated inflammatory biomarkers in patients with obesity, even during pregnancy. The response to periodontal treatment varies, with some improvements seen post-bariatric surgery, though evidence on surgical therapy outcomes is limited. Study heterogeneity and uncontrolled confounding factors limit the generalizability of findings. Further research is needed to understand the underlying mechanisms and develop more effective therapeutic strategies for periodontitis and obesity. Collaboration between periodontal health professionals and obesity experts is essential to moving toward integrated and personalized approaches to managing these interrelated conditions.

## Author contributions

CR-G: Conceptualization, Methodology, Writing – original draft, Writing – review & editing, Investigation, Project administration. JP-V: Writing – original draft, Writing – review & editing, Methodology. NG: Writing – review & editing. AS-R: Methodology, Writing – review & editing. RZ-V: Writing – review & editing. AZ: Supervision, Validation, Writing – review & editing. DS-R: Conceptualization, Funding acquisition, Methodology, Project administration, Supervision, Validation, Writing – review & editing, Investigation.
